# Current status and challenges of percutaneous vertebroplasty (PVP)

**DOI:** 10.1007/s11604-022-01322-w

**Published:** 2022-08-09

**Authors:** Tomoyuki Noguchi, Koji Yamashita, Ryotaro Kamei, Junki Maehara

**Affiliations:** 1 Department of Radiology, National Hospital Organization Kyushu Medical Center, 1-8-1 Jigyohama, Chuo-ku, Fukuoka City, Fukuoka Province 810-8563 Japan; 2 Department of Clinical Research, National Hospital Organization Kyushu Medical Center, 1-8-1 Jigyohama, Chuo-ku, Fukuoka City, Fukuoka Province 810-8563 Japan; 3 Education and Training Office, Department of Clinical Research, Center for Clinical Sciences, National Center for Global Health and Medicine, 1-21-1 Toyama, Shinjuku-ku, Tokyo, 162-8655 Japan

**Keywords:** Osteoporotic vertebral fractures, Percutaneous vertebroplasty, Review, Complication, Perspective

## Abstract

A narrative review regarding percutaneous vertebroplasty (PVP) for osteoporotic vertebral fracture (OVF) is provided herein, addressing the epidemic of OVF in Japan, the latest response to the criticism of PVP for OVFs, the indications and potential risks of PVP for OVFs, and a future perspective for PVP. Each year in Japan, approximately 32,000 patients aged 55 years or older suffer from chronic low back pain for several months to several years due to a compression fracture. PVP is one of the surgical treatments for an OVF, and it is less invasive compared to the traditional open surgery. PVP is suitable for OVF patients who have difficulty walking as assessed by the modified Yokoyama’s activities of daily living (ADL) scoring system, and for patients with Kummell's disease diagnosed by CT and MRI examinations. Serious adverse events related to PVP occur in 1.1–3.3% of the cases, but direct deaths from PVP are extremely rare at less than 1%. Recent studies demonstrated that OVF patients treated with PVP are less likely to die after the treatment than non-surgically treated patients, which conflicts with the Cochran reviews’ conclusion not supporting PVP for OVFs. Novel robotic systems and procedure-support devices are being developed, providing a next step toward fully automated PVP procedures.

## Introduction

Percutaneous vertebroplasty (PVP) is a treatment for patients with one or more symptomatic vertebral fractures caused by a bone tumor, osteoporosis, or trauma. In a PVP, bone biopsy needles are inserted into the fractured vertebra with the patient under local anesthesia; bone cement made of polymethyl methacrylate (PMMA) is injected through the needles, and then symptoms such as walking difficulty or back pain are immediately alleviated [1, 2]. A PVP procedure requires only 2 h of treatment time and 2 h of postoperative bed rest; it can be performed through a 5-mm skin incision for the insertion of each bone biopsy needle, it has a low frequency of serious adverse events, it can be performed without special preoperative preparation or intensive postoperative care, and the only absolute contraindications are an uncontrollable infection and bleeding tendency [2]. The nature of PVP as a minimally invasive procedure with a low rate of complications allows patients to return home after treatment without hospitalization [3] and makes it possible to treat patients over 90 years old and to guarantee therapeutic efficacy [4, 5].

The present narrative review focuses on the following topics: the incidence of osteoporotic vertebral fracture (OVF) in Japan, a response to the controversy regarding the use of PVP for OVFs, assessment and practical indicators to determine PVP indications for OVF patients, PVP complications, features and applications of BKP, and future PVP topics.

## Incidence of osteoporotic compression fractures in Japan

OVFs are common among the elderly. Based on Japan's 2020 population census, the estimated number of OVF patients per year nationwide is 720,000 for those aged 55 years and older and 607,000 for those aged 65 years and older (Appendix 1) [6, 7]. However, about two-thirds of OVF patients are asymptomatic and do not necessarily require intensive treatment. Conversely, about one-third of OVF cases are painful and sometimes require bed rest in a hospital [8]. Patients with an 11th thoracic, 12th thoracic, or 1st lumbar vertebral fracture in the acute phase often require prolonged bed rest because they suffer from severe pain when transitioning from lying to sitting or vice versa. Such long-term bed rest may cause secondary complications; e.g., decreased ambulation, constipation, urinary tract infection, aspiration pneumonia, deep vein thrombosis, and decreased ability to live independently [9].

Nonunion vertebral fractures called ‘Kummell’s disease’ can develop in the chronic phase, and their rate has been reported to reach 13.5% after 6 months from the fracture onset [10]. Based on the above data, it is estimated that each year in Japan, 32,000 patients aged 55 years and over and 27,000 patients aged 65 years and over suffer for several months to several years from chronic low back pain due to pseudoarthrosis after a compression fracture.

For OVF patients, the current treatment policy for asymptomatic patients is based on a no-treatment follow-up or the initiation of treatment for osteoporosis, and for symptomatic patients conservative therapy is recommended [11]. Surgical treatment is used only in cases of nerve compression due to a vertebral fracture or in cases of poor recovery in response to conservative therapy [12]. Considering that OVF is more common in the vulnerable elderly with multiple pre-existing and comorbidities, OVF patients are often not indicated for surgery. It is thus natural that minimally invasive PVP treatment is attracting attention as an intermediate treatment that bridges the gap between conservative treatment and surgical treatment (Table 1).

**Table 1:** Incidence of complications related to PVP

Complication	Incidence
Adverse events	
Serious adverse events related to PVP [3, 48, 49]	1.1–3.3%
Direct deaths from PVP [87]	0.0018–0.002%
Bone cement leakage [3, 24, 48, 49, 51]	34–91.3%
Symptomatic bone cement leakage [79]	1.08%
Venous cement leakage detected by CT [62, 91, 92]	12–26%
Pulmonary cement embolism	
Detection by chest radiograph [62]	1.0–6.8%
Detection by chest CT [62]	2.1–26%
Symptomatic pulmonary embolism [69]	0.40%
Respiratory disorders other than cement embolism [49, 80]	0.2–0.4%
Cardiac cement embolism [70]	3.90%
Symptomatic cardiac embolism [70]	0.33%
Cardiac dysfunction other than cement embolism	
Transient hypotension due to suspected vagal reflex [49]	1.0%
Myocardial infarction [69]	0.05%
Paraplegia/Paraparesis	
Paraplegia [59–61, 89, 90, 94]	< 1%
Paraparesis [59]	0.75%
Nerve root symptoms [59]	0.75%
Vessel damage and bleeding [53, 54, 57–61]	< 1%
Paraparesis due to intracanal subdural hematoma [59]	0.5%
Infection after PVP [69, 72, 74, 75]	0.1–0.46%
Allergy to PMMA [42–44]	< 1%
Puncture in the spinal canal [56]	0.8–4.0%
Vertebral refracture after PVP	
Refracture in adjacent or remote vertebra [3, 24, 48, 49, 69]	4.9–19%
Refracture in treated vertebra [81–83]	0.56–3.2%
Fractures other than vertebral fractures related to PVP [3, 23, 80]	1.1–2.6%
Cerebral infarction [84]	one case report
Fat embolism [85, 86]	two case reports

## The current PVP controversy and latest responses

The use of PVP began in 1987 with the treatment of vertebral hemangiomas by bone cement injection [1]. Due to the PVP characteristics of high safety, high efficacy, and low invasiveness, many patients with OVF have been treated with PVP in the United States since the late 1990s, and the number of research papers concerning PVP surged until 2010. However, it was announced in 2009 that there was no significant difference in therapeutic effects between PVP and a placebo treatment after 6 months [13, 14], and two Cochrane Reviews concluded that PVP treatment for OVFs was not supported [13].

However, it should be noted that the previous studies and reviews not supporting the effectiveness of PVP made a fundamental mistake in drawing these conclusions. Both pain as a sensory phenomenon and the quality of life (QOL) as an emotional perception, which are the main outcomes in the previous randomized control studies, can be evaluated only by self-assessment and are often difficult for both patients and inspectors to quantify accurately. There has been no boycott against PVP around the world; this may be because PVP clearly provides immediate pain relief and a practical improvement in patients' quality of life, along with a very low frequency of adverse events even for elderly patients.

As a latest response to the PVP controversy, its survival benefit addresses the core of the controversy. Significant mortality data should be at the forefront of the conversation regarding the efficacy of vertebral augmentation [15]. Hinde and colleagues recently published a meta-analysis of more than 2 million patients, including 16 studies in the US and 6 studies in other countries. The meta-analysis demonstrated that OVF patients treated with PVP or balloon kyphoplasty (BKP) had a 22% lower chance of dying up to 10 years after the treatment compared to those who received non-surgical treatment [16]. In another article analyzing BKP and PVP utilization for vertebral compression fracture patients in the US Medicare data set from 2005 to 2014, BKP and PVP had a 19% and 7% lower propensity-adjusted 10-year mortality risk than non-surgical treatment, respectively [17, 18]. These survival benefit findings counteract the criticism regarding the efficacy of vertebral augmentation that arose after the 2009 ‘sham’ control studies and the Cochran reviews' lack of support for performing PVP for patients with OVFs [15, 17, 19].

In Japan, in contrast, the application of PVP for OVFs has not become widespread even though the PVP procedure has been covered in part by the country’s national health insurance program since 2012. The reasons for this are as follows: First, the bone cement is surprisingly indicated only for neoplastic vertebral fractures but not for osteoporotic vertebral fractures. Strategic ideas aiming for the Japanese regulatory approval may be needed such as a new medical device kit for the bone cement along with bone biopsy needles like the BKP medical device kit. Second, the indication criteria for PVP are not well known. It is easier to understand if the vertebral fracture is considered separately for the acute vertebral fracture and Kummell’s disease, which is described later. Third, there are not many experts who can perform PVP. Appropriate training meetings for PVP procedures should be encouraged, together with full PVP insurance coverage. In light of Japan’s ‘super-aging’ society, the above problems will be hopefully solved in order to prevent the poor prognoses of elderly who need long-term care due to vertebral fractures.

## Assessment of the pathological state of OVF patients

Once elderly people incur an OVF, they usually undergo the medical assessment of their back pain and QOL. Pain assessments are generally performed by using a numerical rating scale (NRS) or a visual analog scale (VAS). An NRS is used to estimate a patient's pain by having the patient choose a number in the range 0–10 where 0 indicates no pain and 10 indicates the maximum possible pain. A VAS is a 10-cm line with the words “no pain” on one end and “worst pain ever” on the other, and the patient is instructed to show a point on the line that represents the patient’s perception of his or her current condition [20]. Investigations of the QOL of patients with vertebral fracture have used the following: a Medical Outcome-Short Form (SF-36) [21], the Oswestry Disability Index (ODI) [22], the Quality of Life Questionnaire of the European Foundation for Osteoporosis (QUALEFFO-41) [23], and Roland-Morris Disability Questionnaire (RMDQ) [3, 24].

However, as mentioned above, pain and quality of life are highly dependent on a patient's subjective perceptions. In the medical community, it is known that the satisfaction a patient feels after receiving a treatment that is valuable plus a good relationship with the treating medical staff are effective in providing the pain relief and a better QOL for the patient. In contrast, the circumstances that caused a patient's OVF, treatment-related bad feelings, anxiety about the future, and/or worsening relationships around the patient can contribute to sustained pain and even a worsening of his or her quality of life [25, 26]. Self-assessment indices should be used only as a reference when deciding on the indications for PVP and patient monitoring after PVP.

## Indications and contraindications of PVP based on the characteristics of OVF

**Fig. 1:**
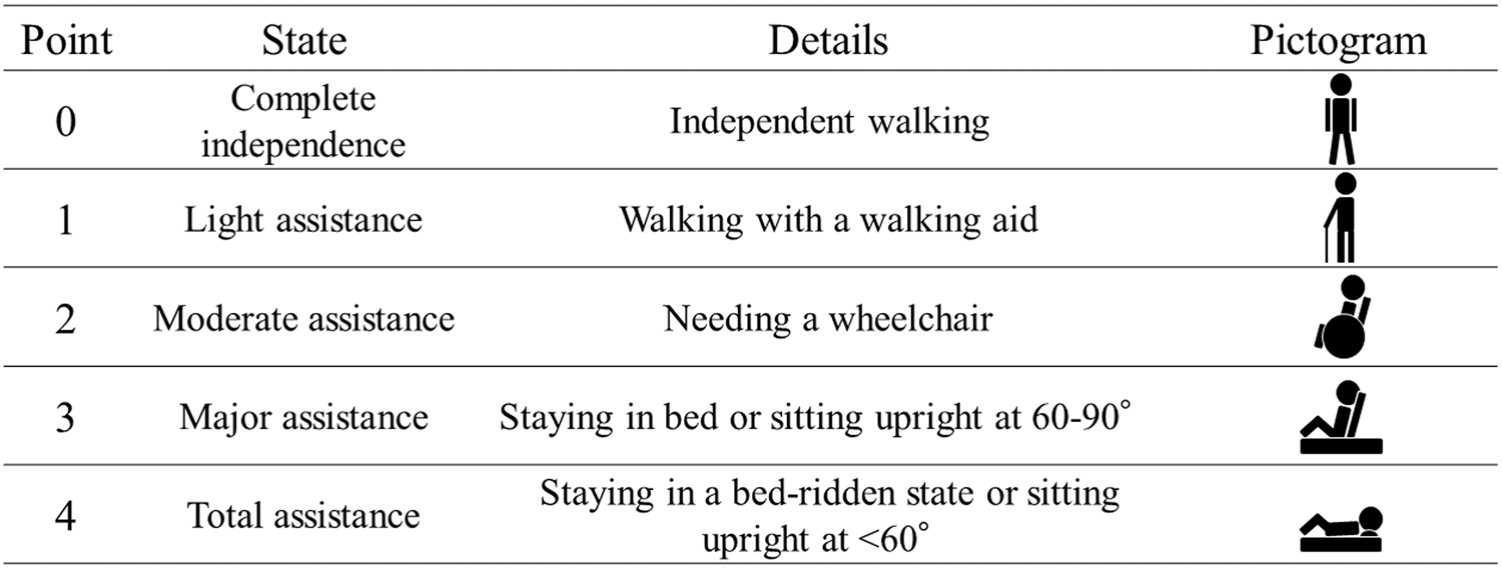
The modified Yokoyama’s ADL scoring system is a numerical value from 0 to 4 that objectively and simply expresses mobility in bedridden, sitting, transferring, and walking after an OVF

While both pain and QOL are subjective no matter what measurement system is adopted, the modified Yokoyama's activities of daily living (ADL) scoring system, which is an ADL measurement, is noteworthy [27, 28]. This system is as follows (Fig. 1): 0 points = complete independence, independent walking; 1 point = light assistance is needed, walking with a walking aid; 2 points = moderate assistance, needing a wheelchair for locomotion; 3 points = major assistance, mostly staying in bed or sitting upright at 60°–90°; 4 points = total assistance, mostly staying in a bed-ridden state or sitting upright at less than 60°. This scoring system has several advantages, including ease of use, simple and objective estimates by any medical staff, and a direct relationship between the mobility scores and the physical condition of patients with vertebral fractures. Clinical problems are also easy to predict with this system. For example, patients who score 3 or 4 points usually stay in bed, urinate/defecate in a bedridden state, require frequent medical staff assistance, and are at increased risk of secondary illness and long-term hospitalization. Patients with 2 points are normally in a wheelchair, urinate and defecate in a bathroom, undergo advanced rehabilitation for walking but still need assistance, and go on limited outings. Patients with 0 points or 1 point can walk, return home, and expand their activities [27].

**Fig. 2:**
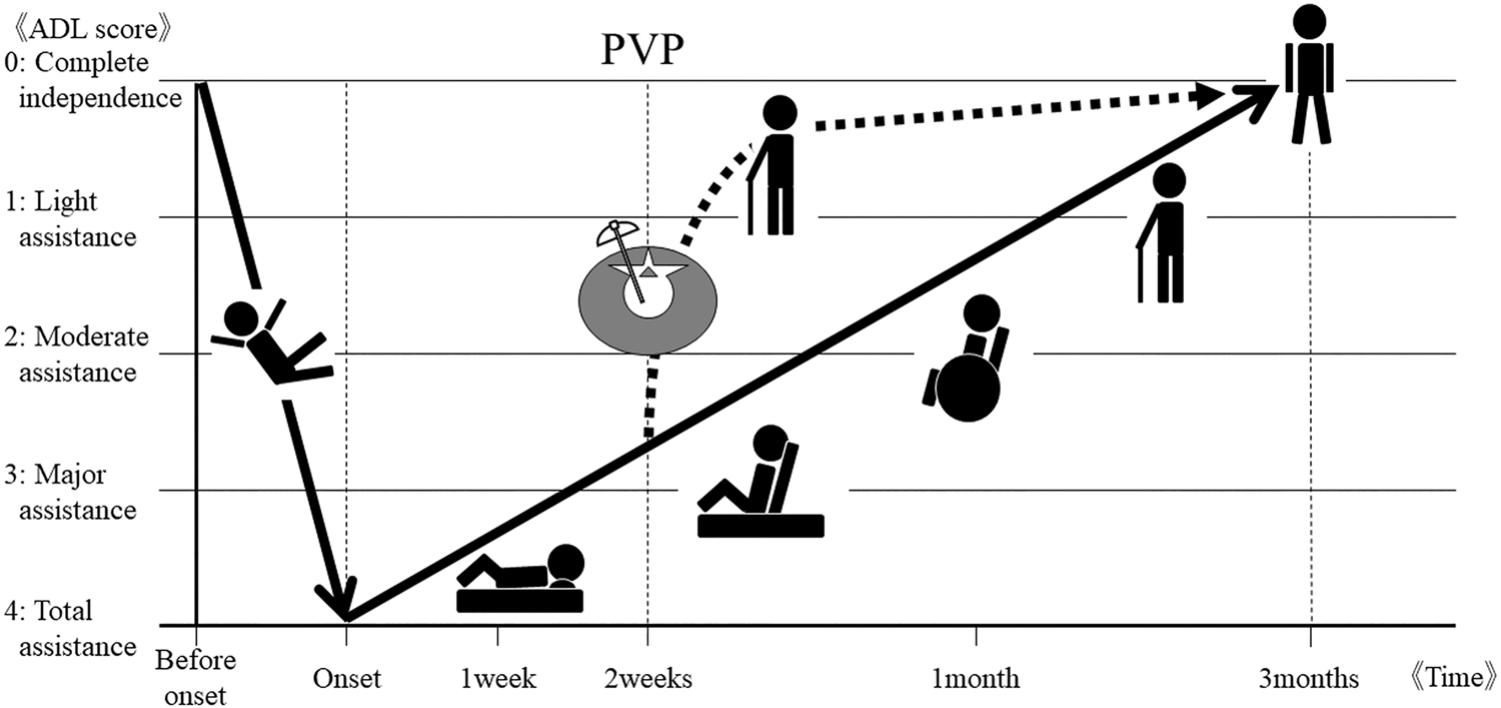
A hypothesis regarding the relationship between vertebral fractures and PVP. When an OVF significantly reduces a patient's QOL, most patients gradually recover with conservative therapy in 3 months, as shown by the solid black line. Patients are expected to recover rapidly with PVP intervention (black dotted line). A PVP can help a patient achieve early recovery and early bed leaving, especially for patients who are at risk of cognitive decline due to debilitation or to aspiration pneumonia or delirium due to long bed rest

Figure 2 illustrates the temporal mobility change after an OVF due to a fall, which is common situation for the occurrence of an OVF. The vertical axis is the modified Yokoyama's ADL scoring system and the horizontal axis is the time course. After the patient's mobility becomes extremely low due to the vertebral fracture, in most cases, it may gradually recover in approximately 3 months with conservative treatment (as shown by the solid black line in Fig. 2) [29]. If the patient undergoes PVP, he or she is expected to have a rapid recovery (the dotted black line). PVP is positioned as a first-line treatment to achieve early recovery and early out-of-bed status, especially for such patients who are old enough for there to be concern about weakness, aspiration pneumonia, delirium, and/or cognitive decline due to prolonged bed rest. Thus, the modified Yokoyama's ADL scoring system is an excellent indicator for objectively determining PVP indications and for assessing post-PVP effects.

On the other hand, there are also cases in which low back pain persists (i.e., Kummell's disease) in which the healing of trivial vertebral fractures or bone bruises is prolonged and nonunionized [30]. Kummell's disease is thought to be caused by the avascular osteonecrosis of the vertebral body, but the detailed pathogeny is unknown [31]. Kummell's disease is objectively diagnosed by imaging examinations that prove gas or fluid retention in the cleft inside the vertebral body [32]. Patients with Kummell's disease may be treated by non-surgical interventions including bed rest, lumbar traction, a brace, analgesics and anti-osteoporosis drugs, all of which may not be sufficient and may be ineffective. Moreover, these interventions might pose the risk of a delayed spinal collapse causing paralysis [33, 34]. Thus, patients with Kummell's disease without neurological symptoms related to spinal cord compression may be good candidates for PVP, whether or not they are able to walk [35–37]. PVP has been shown to be able to immediately relieve low back pain in Kummell's disease, achieve satisfactory clinical efficacy, partially restore vertebral height, and correct kyphosis [34, 38, 39].

Ambulatory OVF patients with no Kummell's disease have shown limited benefits of PVP and should be treated conservatively. However, Venmans et al. reported that while 40% of conservatively treated patients had sufficient pain relief during the first 3 months post-fracture onset, the other 60% still had pain at the last follow-up at 12 months. Patients who have low back pain for more than 3 months due to an unhealed vertebral fracture can thus be considered candidates for PVP [29].

Clinicians should also be aware of the exclusion criteria for PVP as proposed in the guidelines for PVP for osteoporotic vertebral fracture [40, 41]; (1) uncontrollable local or systemic infections, (2) uncontrollable bleeding tendency, (3) allergies to bone cement or opacification agents, (4) back pain from a condition other than a vertebral fracture, (5) major organ dysfunction, (6) under 55 years of age, (7) difficulty in the prone position, (8) 4 or more vertebral fractures, and (9) vertebral posterior wall damage. (1)–(3) and (4)–(9) are considered to be absolute and relative contraindications to PVP, respectively.

PVP for a patient with infectious spondylitis or an uncontrollable systemic infection should be avoided because the removal of bone cement is difficult when the activation of an infection occurs. PVP is one of the nonvascular procedures and involves less bleeding compared to other vascular-approach procedures. However, a bleeding tendency should be controlled, as there is no guarantee that vessels will never be accidentally punctured during the procedure. Allergy to bone cement or opacification agents is reported to be very rare [42–44], and is an absolute contraindication to PVP.

PVP is not effective for back pain other than vertebral fractures such as nerve compression pain, because PVP is effective by stabilizing the vertebral body with bone cement injected into the cleft of the fracture [45, 46]. However, nerve compression pain that coexists with vertebral fracture pain might be indicated for PVP because nerve compression caused by spinal instability may be reduced by PVP. Major organ dysfunctions are relatively contraindicated. In particular, patients with a substantial amount of pleural effusion can find it difficult to tolerate the prone position for the approximately 2-h period that is necessary to perform PVP. Patients under the age of 55 years are also relatively unsuitable for PVP indications because the bone cement is permanently placed and difficult to remove. Difficulty in the use of prone position is thus one of the relative contraindications for PVP. The use of the lateral decubitus position for PVP is possible [47], but technically difficult. The PVP for patients with four or more spinal fractures may be accomplished in two or more sessions, because the safety of the in vivo use of two or more packs of bone cement preparation is not guaranteed. In cases of a vertebral fracture with posterior wall damage, close attention should be paid to the possibility of bone cement leakage into the spinal canal. In patients with a history of thoracic or lumbar spine surgery, difficulty in obtaining visibility on fluoroscopy during the procedure may be encountered.

**Fig. 3:**
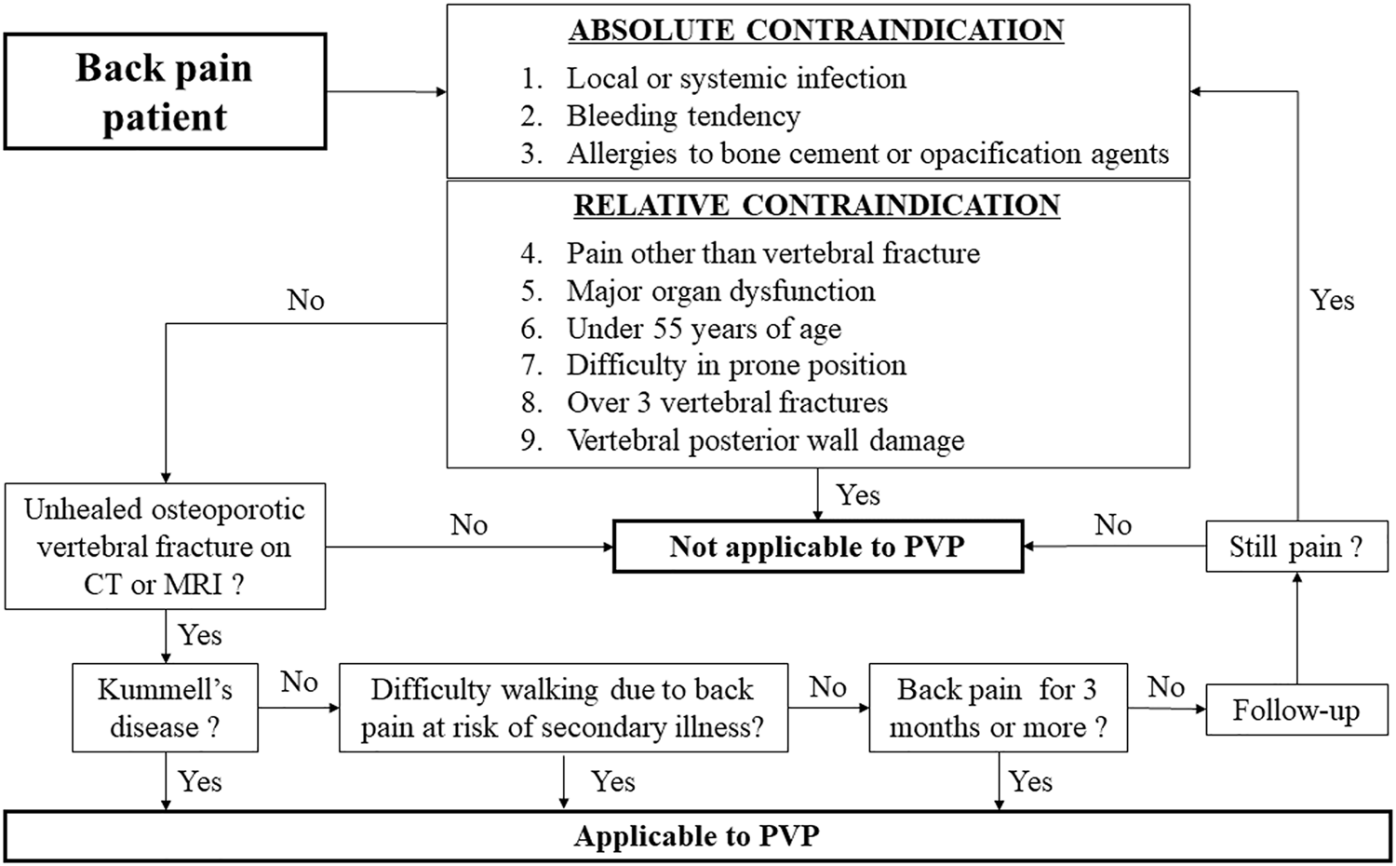
Clinical reasoning flowchart of PVP indications for low back pain. Symptomatic OVF patients without an uncontrollable local or systemic infection, uncontrollable bleeding tendency, allergies to bone cement or opacification agents, major organ dysfunction, under 55 years of age, with difficulty in being in the prone position, more than 3 vertebral fractures, or vertebral posterior wall damage may be eligible for PVP if they have Kummell's disease, difficulty walking due back pain at risk of secondary illness, or back pain that lasts more than 3 months

Figure 3 is a flowchart based on the above information. First, the presence of an unhealed OVF on CT or MRI should be confirmed in a patient with low back pain, and the patient's condition should not meet any of the PVP exclusion criteria. The patient may be eligible for PVP if he or she has Kummell's disease, difficulty walking due to back pain at risk of secondary illness, or back pain that lasts more than 3 months.

## Potential risk of PVP procedure for osteoporotic vertebral fractures

It must be acknowledged that like essentially every medical procedure, PVP is not perfectly safe [3, 24, 48–52]. Adverse events of PVP include damage to the spinal cord and thoracoabdominal organs or bleeding due to inappropriate puncture needle insertion [53–61], compression or damage to the spinal cord or nerve roots, venous thrombosis, or cardiopulmonary cement embolization due to leakage of bone cement outside the vertebral body [3, 24, 42–44, 48, 49, 51, 55, 59, 62–70], sudden reduction in blood pressure and shock symptoms due to allergic reactions [42–44], infection due to a non-sterile operation [69, 71–75], vertebral refractures or fractures other than in a vertebra [3, 23, 24, 48, 49, 69, 76–83], and the other rare but serious complications [49, 69, 80, 84–86]. This section describes notable complications in conjunction with their incidence even if they occur very rarely.

### Incidence of all adverse events

Symptomatic adverse events related to PVP occur in 1.1–3.9% of cases [3, 41, 48, 49], but the patients have almost always recovered. Direct deaths from PVP are extremely rare at 0.0018–0.002% [87]. However, OVF patients who do or do not undergo PVP are at a 1.2–5% risk of death from another disease within 1 year after fracture onset [3, 24, 48, 49, 51, 52]. The 10-year risk of death for OVF patients was reported to be surprisingly high at 85.1% [17]. It should be noted that even though PVP is a low-risk treatment, OVF patients are at high risk of dying from diseases other than osteoporosis.

### Bone cement leakage

Bone cement leakage has been observed in 34–91.3% of vertebral bodies [3, 24, 48, 49, 51]. However, most of these cases are asymptomatic. Lee et al. reported in their meta-analysis that symptomatic cement leakage occurs in 1.08% [79]. Complications associated with bone cement leakage include pulmonary cement embolism [62, 69, 88], cardiac cement embolism [63, 70], bilateral lower limb paraplegia due to intravertebral canal cement leakage [55, 59, 89, 90], and a cement-embolic cerebral infarction [84], which are described below.

### Pulmonary cement embolism (PCE)

The reported incidence of pulmonary cement embolism (PCE) in PVP cases was 1–6.8% as shown by chest radiographs and 2.1–26% revealed by chest computed tomography (CT) [62]. However, a PCE is almost always asymptomatic; symptoms associated with a PCE were observed only in 0.4% of PVP cases [69]. Patients with a PCE can be divided into four groups; those with a (1) asymptomatic peripheral embolism, (2) symptomatic peripheral embolism, (3) asymptomatic central embolism, and (4) symptomatic central embolism (central embolism includes the main pulmonary trunk and / or right or left main pulmonary artery, and an embolism beyond that is considered a peripheral pulmonary embolism). For group 1 patients, no treatment is recommended. For groups 2 and 3 patients with a symptomatic peripheral or asymptomatic central embolism, it is recommended that standard treatment guidelines for the treatment of thrombotic pulmonary embolism should be followed. For group 4 patients with a symptomatic central embolism, surgical treatment by embolectomy has been proposed [62].

### Respiratory disorders other than PCE

The exact frequency or respiratory disorders other than PCE is at 0.2–0.4% [49, 80]. Kobayashi et al. reported the case of a PVP patient with a transient decrease in oxygen saturation during treatment due to a suspected sedative overdose [49]. Two respiratory arrest cases have also been reported [80]. There were no deaths as a result of PVP in either report.

### Cardiac cement embolism

Venous cement leakage of patients treated with PVP occurs in 12–26% detected by CT [62, 91, 92], and a cardiac cement embolism may further migrate to the right ventricle and pulmonary artery [63]. The reported incidence of intracardiac cement embolism after PV was 72/1854 sessions (3.9%), of these, only 5 cases (0.33%) were symptomatic [70]. The most common symptoms appear to be chest pain and dyspnea. There is a case report of PMMA leaking from a vein moving to the heart and piercing the heart, but the patient recovered after emergency surgery [93].

### Cardiac dysfunction other than cement embolism

Transient hypotension due to suspected vagal reflex was reported in five of 485 patients (1.0%) during PVP [49]. The rate of myocardial infarction was one in 1,938 (0.05%) PVP cases [69]. There were no deaths as a result of PVP in either of these reports.

### Neurological damage related to PVP

Paraparesis and nerve root symptoms after PVP are each estimated to occur in approximately 0.75% of cases, respectively [59]. Paraplegia or paraparesis is reported to be caused by spinal canal stenosis due to bone cement leakage, a migration of bone cement to anterior spinal artery, an intracanal hematoma associated with needle puncture, or an unknown cause. To prevent these complications, penetration of the inside of the pedicle should be avoided, and the viscosity of the PMMA must be monitored. Immediate spinal canal decompression surgery is recommended when paraplegia is due to a space-occupying lesion in the spinal canal [59–61, 89, 90, 94].

### Vessel damage and bleeding

There are reports about incorrect punctures of the thoracoabdominal aorta and lumbar artery [53, 54, 57–59]. Such punctures can also cause intracanal bleeding (extradural, subdural, subarachnoid hemorrhage), which can lead to transient lower limb paralysis or sequelae [59–61]. Paraparesis due to the intracanal spinal epidural hematoma were reported to be 0.5% [59].

### Post-PVP infections

Infectious complications after PVP are extremely rare at 0.1–0.46% [69, 72, 74, 75]. However, in PVP patients with significant neurological symptoms, severe instability due to an infected fracture, or resistance to antibiotics, prompt surgical treatment should be considered. Risks of infection are suspected to be associated with a history of bacteremia, urinary tract infection, or pulmonary tuberculosis prior to PVP [72, 74, 75]. At least for the prevention of perioperative infections, prophylactic antibiotics during a PVP procedure are recommended [95].

### Allergy to PMMA

The exact frequency of allergy to PMMA is unknown, as it is rarely reported or summarized; the incidence is probably less than 1%. PMMA is commonly used for medical products such as dialysis membranes, intraocular lenses, dental fillers, and more. PMMA allergic reactions are thus extremely rare. However, it should be noted that cases of an acute or delayed allergic reaction to PMMA (including death) have been reported [42–44].

### Puncture into the spinal canal

The rate of a puncture-needle penetration into the spinal canal during PVP is 0.8–4.0% [56]. All of the reported patients with a puncture-needle penetration into the spinal canal were asymptomatic with conservative treatment of extended postoperative resting time and follow-up by imaging examinations. Even if an experienced PVP expert administers the needle punctures, he or she cannot accomplish zero penetration into the spinal canal. CT images should be obtained immediately after the puncture(s), and the path of the puncture needle should be checked; if the needle penetrates the spinal canal, the leakage of PMMA into the spinal canal must be prevented, and a careful follow-up after PVP for secondary complications such as cerebrospinal fluid leakage and intracanal bleeding should be conducted.

### Refracture in adjacent or remote levels of the vertebra after PVP

A vertebral refracture occurs in 4.9–19% of patients after PVP. However, the frequency of vertebral refracture after PVP is equal to that without PVP. Vertebral refractures after PVP can also occur in adjacent as well as distant vertebral bodies [3, 24, 48, 49, 69], but, empirically, patients who are re-treated for recurrence of compression fractures a few days after PVP have often new compression fractures of adjacent vertebral bodies directly above or below the PVP-treated vertebral body [96]. Prophylactic PVP as an optional treatment is preferably performed on normal upper and lower vertebral bodies adjacent PVP-treated vertebra [97, 98].

### Refracture of PVP-treated vertebra

In rare cases at 0.56–3.2%, PVP-treated vertebral bodies may re-collapse and symptoms may recur [81–83]. In particular, lumpy cement may move within the vertebral body [99], resulting in incomplete compression fracture healing. In such cases, PVP should be re-executed and the movable cement should be fixed to the vertebral body with additional bone cement, or a corpectomy in conjunction with posterior fixation and internal fixation should be performed. In order To prevent such cement movement, it is desirable to perform PVP as well as a pediculoplasty in which cement is placed along the pathway of the needle within the vertebral arch as an anchor. Experimentally, there was a case in which the anchor broke and started to move the cement during the patient's activities of daily living when the pediculoplasty was performed with a 13-gauge needle. Therefore, for post-PVP patients with Kummell's disease, in which lumpy cementation can be expected, a pediculoplasty with an 11-gauge needle is considered desirable.

### Fractures other than vertebral fractures related to PVP

In rare cases at 1.1–2.6% [3, 23, 80], fractures other than compression fractures occurring during PVP including rib fractures [41, 80, 100], humerus fractures [3], and transverse process fractures [80] have been reported. Careful PVP treatment for patients with osteoporosis is essential. As a pitfall, the new symptoms that appear after PVP may actually be due to preexisting fractures elsewhere. Some patients with severe back pain may not be aware of relatively mild pain in another part of the body. A careful preoperative assessment and a review of pre- and postoperative imaging examination may be informative.

### Cerebral infarction

A cerebral infarction was reported in a 71-year-old woman who had a stroke ~ 30 min after the completion of PVP. Bone cement migrated into the venous system, resulting in a left middle cerebral artery embolus. The patient was presumed to have a right-to-left shunt from either a patent foramen ovale or a pulmonary arteriovenous malformation [84].

### Fat embolism

A fatal case with fat embolism and a cerebral embolism case with fat embolism were reported. Fat embolism is considered rare, but clinicians should be aware of it [85, 86].

## Features and applications of BKP

Whereas PVP aims to stabilize vertebral fractures by percutaneously injecting bone cement into the vertebral body, BKP not only stabilizes the vertebral body but also restore body height, which may be thought as an advanced method of PVP [101]. BKP, which was first clinically applied to patients with OVFs by Lieberman et al., restores vertebral body height by inflating a balloon inserted into the vertebral body to form a cavity and injecting bone cement into the vertebral body [102]. As a secondary advantage, BKP has a low frequency of cement leakage. This is because BKP allows low-pressure cement injection into the cavity, whereas PVP requires high-pressure cement injection into the vertebral body with residual trabecular structure [103]. In meta-analyses of clinical studies, BKP indeed restored higher vertebral body height with less frequent cement leakage compared to PVP. However, cement leakage usually did not cause clinical symptoms, and radiographic differences of vertebral height restoration did not significantly affect clinical outcomes. That is, both BKP and PVP were effective in reducing pain, improving ODI, and controlling major complications, without any significant difference between the two [104, 105]. Also, regarding the hypothesis that recovery of kyphosis promotes recovery of respiratory function, both BKP and PVP improved respiratory function in patients with chronic obstructive pulmonary disease (COPD) with no significant difference between the two although BKP had higher vertebral height recovery than PVP [106]. We should reconfirm the fact that PVP has a considerable effect of vertebral body height restoration, where BKP is significantly superior to PVP [107]. On the other hand, regarding the hypothesis that BKP's bone cement forms a solid lump which reduces the elasticity of the bone and induces refracture of the adjacent vertebral body [76], there was no significant difference of the vertebral refracture rate between the two [108].

The cost of BKP is 3 to 4 times that of PVP [108, 109]. BKP requires medical equipment such as an inflatable bone tamp in addition to bone biopsy needles and bone cement, and BKP is usually performed under general anesthesia and requires hospitalization for at least one night [101]. On the other hand, PVP is usually performed with local anesthesia, requires only bone biopsy needle and bone cement, and outpatient treatment is also possible [3].

Heini et al. proposed the indication of BKP as follows [101]; (1) patients with osteoporotic vertebral fractures whose height loss is related to a spinal stenosis and height restoration can relieve the symptoms, (2) patients with traumatic fractures where the endplate relocation should be attempted, or (3) patients with bone tumor for whom the cavity formation might help with difficulty of tumorous lesions.

## Future advancement of PVP

Novel robotic systems and procedure-support devices are being developed, providing a next step in fully automated PVP procedures. Neumann et al. developed a robotic system that can be remotely controlled to inject bone cement, along with a cold passive exchanger that slows cement hardening [110]. A navigation system in which a C-arm tracker, patient tracker, and puncture-needle tracker coordinate spinal imaging information to assist in accurate punctures in PVP was described by Xu et al. [111]. Wang et al. proposed a method to guide the puncture in BKP that uses a system consisting of a reference tracker, robotic arm, and monitor [112]. As a unique attempt, a 3D-printer was used to create a puncture guide adapter for PVP with two sockets for needle insertion [113].

As an advanced computing system applied to PVP, Uchiyama et al. developed a 'spinal needling intervention practice using ray-summation imaging' (SNIPURS) to assist radiology trainees to learn needle puncturing [114]. Trainees freely orient the workstation-generated spinal ray-summation image to view the appearance of the spine in any direction by using a computer mouse, and they can then determine the on-end puncture point in the selected direction. As the post-processing, the virtual needle is visualized with an excavation tool, and the path of the puncture needle is evaluated on the workstation. Trainees undergo a total of 48 vertebral needle targeting simulations and complete basic PVP procedures. The above-described equipment and methods are promising, and further developments are expected.

## Summary

In Japan, each year roughly 32,000 patients aged 55 years or older are estimated to suffer from low back pain for several months to several years due to pseudoarthrosis after experiencing a compression fracture. PVP is a treatment that can provide immediate pain relief and mobility improvement from difficulty in sitting, transfer, and walking due to vertebral fractures. PVP is also suitable for patients without neurological deficit but with Kummell's disease, which is diagnosed by imaging examinations that confirm gas or fluid retention inside the vertebral body. Serious adverse events related to PVP occur in 1.1–3.3% of cases, and direct deaths from PVP are extremely rare (less than1%). Recent reports demonstrated that OVF patients treated with PVP or balloon kyphoplasty had a lower chance of dying within 10 years post-fracture onset than those who received non-surgical treatment. This is in opposition to the Cochrane Reviews' conclusion that vertebral augmentation for osteoporotic vertebral fractures is not supported. Both BKP and PVP are effective in improving the pain and QOL caused by OVF with minimal complications. Advanced robotic systems, procedure-support devices, and computing training systems are promising, and further PVP developments are expected.

## Appendix

The 10-year cumulative incidence of compression fractures in men and women with absence of vertebral fractures at the initial survey is 2.2% and 2.1% for 40–49 years of age, 4.9% and 4.5% for 50–59 years, 6.1% and 14% for 60–69 years, and 10.8% and 22.2% for 70–79 years, respectively [6]. From these findings, the average annual incidence is calculated to be 0.222% and 0.212% for 40–49 years, 0.501% and 0.459% for 50–59 years, 0.522% and 1.497% for 60–69 years, and 1.136% and 2.479% for 70–79 years, respectively. Applying this to Japan's 2020 population census [7] with assumption that the one-year incidence of aged 80 and over is same as 70–79 years, the incidence of compression fractures aged 55 and over was 720,000, and the incidence of compression fractures aged 65 and over was 607,000. While many of these patients are asymptomatic, 33% of them develop symptomatic compression fractures [8]. Accordingly, the incidence of symptomatic compression fractures aged 55 and over was 240,000, and those aged 65 and over was 203,000. 13.5% of these patients develop nonunion and chronic low back pain after 6 months [10]. The nonunion rate after compression fractures over 55 years was 32,000, and those aged 65 and over was 27,000 in Japan.
